# Supplementary material for: Smart scanning for low-illumination and fast RESOLFT nanoscopy in vivo
Source: Nat Commun. 2019 Feb 1;10:556. doi: 10.1038/s41467-019-08442-4 (PMC6358637; doi:10.1038/s41467-019-08442-4)
Supplement: Supplementary file 1 — Supplementary Information [file 41467_2019_8442_MOESM1_ESM.pdf]

Supplementary information

for

Smart scanning for low-illumination and fast RESOLFT nanoscopy *in vivo*

Jes Dreier et al

Supplementary Note 1

Quantification of the scanning accuracy and spatial resolution under different probe types in smart RESOLFT imaging

We compared smart RESOLFT to regular RESOLFT imaging. As shown in (Figure 2) we investigated whether the smart scanning mode caused any structures to be skipped, i.e. false negative. This included a large data set of 72 different regions, in 3 different samples. It was found that smart RESOLFT did not cause the loss of any structure, as long as the decision value was set at the right level (1-3 photons). Secondly, we did a quantitative comparison of the spatial resolution of both regular RESOLFT and the four different illumination pulse schemes for smart RESOLFT imaging. We compared a large series of FWHM values obtained by Lorentzian fits to line profiles data measured on single Vimentin filaments. Each filament was averaged on 3 consecutive pixels to minimize noise effects. We compared the median value, as an alternative to the mean value, since the median is less affected by outliers. As can be seen (Supplementary figure 1a) the median gives very similar values and a t-test shows no significant difference between them.

As with any super resolution technique, it is worth considering what level of spatial resolution is needed for a given experiment. With RESOLFT we have an additional variable that controls the spatial resolution aside from the usual intensity, namely the OFF-switching time, i.e. how long the OFF-switching laser spend in each pixel. Reduced OFF-switching time can greatly increase the scan speed, and thereby minimize the overall frame time. We measured the resolution as a function of the OFF-switching duration (Supplementary figure 1b). As expected the spatial resolution increases for longer OFF-switching pulse duration until a plateau determined by the signal to noise ratio of the few rsFPs left in the ON state. The data have been fitted to a variation of the STED resolution equation.

$$D = \frac{\lambda}{2NA} \cdot \frac{1}{\sqrt{1 + \frac{I \cdot t_{off}}{I \cdot t_0}}} = k_1 \cdot \frac{1}{\sqrt{1 + k_2 \cdot t_{off}}} \quad \text{(Eq. 12)}$$

With k_1 and k_2 being constants. The equation fitted reasonably well with the measured resolution values. The graph was used to estimate the needed OFF pulse duration for the measurement of the Pex-16-rsEGFP2 labeled peroxisomes (Figure 5). Based on the literature[1], we decided a resolution of about 100 nm was enough to resolve the individual peroxisomes, and therefore an off-time of 250 µs was chosen.

Supplementary Note 2

Structures occupancy and decision-making

In our approach the increase in recording speed is not constant but specimen-adaptive. Intuitively the less pixels covered by structures in the field of view the faster scan speed will be obtained. However, it does matter how the different structures are distributed in the field of view because the illumination probe is done with beams of a certain physical size (Supplementary figure 1c). Thus, we investigated how the increase in the recording speed depends on the different degree of structures occupancy of the field of view, or in other words, on the sparsity of the sample. The results are plotted in graphs (Supplementary figure 1d-e) and they are built on the analysis of the vimentin images shown in (Figure 2). We plotted the increase in recording speed as function of the percentage of skipped pixels (Supplementary figure 1d). As expected this leads to a very even curve, showing us that the overall frame time is a direct relationship between the imaged and skipped pixels. The different probe types give rise to slightly different graphs, due to the different illumination pulse scheme with probe type 1 being the fastest (decision time 40µs) and probe type 3 being the slowest (decision time

150µs). However, it is interesting to compare the increase in speed with the actual biological structure (Supplementary figure 1e). The biological structures were measured by applying a fluorescence intensity threshold on the regular RESOLFT image. The data show a similar trend but with a much larger variation, because different biological structures gives rise to different degrees of skipped pixels. The relationship between the probe types is maintained, with probe type 1 being the fastest, and also the one with the shortest decision time (40 µs). Probe type 3 is again the slowest, both because it has the longest decision time (150 µs), but also because it uses a bigger PSF, and as such less pixels can be skipped compared to the other probes, as is also illustrated in the scheme (Supplementary figure 1c).

Supplementary Note 3

Temporal Evolution of the RESOLFT Point-Spread-Function

The point-spread-function (PSF) of a RESOLFT microscope is not only a function of space, but also a function of time. In particular, the region from which the fluorescence is collected changes as a function of the time t, where t = 0 states the beginning of the RESOLFT cycle within the pixel. Here, as example, we describe the temporal evolution of the PSF of the RESOLFT microscope when implementing the probe type 2. Similar pictures can be derived for all other probe types.

To describe the temporal evolution of the PSF we used a simplified model, where (i) the proteins can be in two states, the ON and OFF states; (ii) the population of the ON state at the beginning of each RESOLFT cycle (i.e., each pixel) is null; (iii) fluorescent emission occur only when the protein is in the ON states; (vi) transition from the OFF to the ON states occurs with a rate which is linearly proportional to the intensity of the ON-switching beam (405 nm); (v) transition from the ON to the OFF states occurs both during the OFF-switching process and during the fluorescence read-out process with rates linearly proportional to the intensities of the OFF-

switching and read-out illumination beams at 488 nm. Under these conditions the population of the ON (P_{ON}) and OFF (P_{OFF}) states during a RESOLFT cycle including an ON-switching step ($0<=t<T_{ON}$), a read-out step for the probing ($T_{ON}<=t<T_{read1}$), an OFF-switching step ($T_{read1}<=t<T_{OFF}$), and a read-out step for imaging ($T_{OFF}<=t<T_{read2}$), read

$$P_{OFF}(t) = \begin{cases} 1, & t = 0 \\ P_{OFF}(0)exp\left(-I_{ON}/I_{sat\_ON}\right), & 0 < t \leq T_{ON} \\ 1 - P_{ON}(t), & T_{ON} < t \leq T_{read2} \end{cases} \quad \text{(Eq. 1)}$$

and

$$P_{ON}(t) = \begin{cases} 1 - P_{OFF}(t), & 0 \leq t \leq T_{ON} \\ P_{ON}(T_{ON})exp\left(-I_{read1}/I_{satRead1}\right), & T_{ON} < t \leq T_{read1} \\ P_{ON}(T_{read1})exp\left(-I_{OFF}/I_{sat\_OFF}\right), & T_{read1} < t \leq T_{OFF} \\ P_{ON}(T_{OFF})exp\left(-I_{read2}/I_{satRead2}\right), & T_{OFF} < t \leq T_{read2} \end{cases} \quad \text{(Eq. 2)}$$

where I_{sat_ON} is defined as the intensity to reduce 50% the population of the OFF state during the ON-switching step, $I_{satRead1}$ as the intensity to reduce 50% the population of the ON state during the probe step; I_{sat_OFF} as the intensity to reduce 50% the population of the ON state during the OFF-switching step; $I_{satRead2}$ as the intensity to reduce 50% the population of the ON state during the read-out step. Similar Equations can be derived for the other RESOLFT schemes proposed in the main text.

We show the population of the ON state as a function of space and time during the four different steps of the RESOLFT cycle (Supplementary figure 2**)** for the probe type 2, i.e., Gaussian-shaped ON-switching, probe and readout focal intensity distributions and doughnut-shaped OFF-switching intensity distributions. The temporal PSFs of the RESOLFT system for the probe, OFF switching and read-out step are obtained considering the fluorescent emission

proportional to the population of the ON states and to the focal intensity distributions of the respective beams. Cumulative (across the time) PSFs during the three steps (probe, OFF-switching and read-out) are also reported. These are the effective PSFs associated to the images obtained during the smart-RESOLFT measurements. The focal intensity distributions of the different beams to calculate the PSFs are obtained using a fast-Fourier transformation method[2] and assuming: (i) an oil 1.4 NA objective lens; (ii) a pinhole of 1 A.U.; (iii) an ON-switching wavelength of 405 nm; (iv) a probe, OFF-switching, and read-out wavelength of 488 nm; (v) a fluorescent emission at 520 nm.

Supplementary Note 4

Quantification of the Spatial Resolution in the decision map

During the probe pulse the rsFPs emits fluorescence photons, which are used to make the decision on whether to skip or to image a certain region in the sample. They are classified in the first di_i detection window named r1 (Figure 1d). Experimentally we found that the image reconstructed by these photons shows higher spatial resolution than a conventional confocal image. The probe image is recorded by two shorted pulses of 405 nm (20 us) and 488 nm (50 us) focused light.

The conventional confocal is instead recorded with no OFF switching and with a longer ON switching pulse (50 us) followed by a blue 488 nm pulse (50 us). The longer ON switching pulse is applied to ensure that most of the rsFPs are driven into the ON state and then read-out. Importantly, no OFF-switching pulse is applied in this configuration.

By comparing the probe and the confocal images of vimentin filaments we found different spatial resolution. The filaments width is smaller for the structure in the probe image. The median values of the filament’s width distribution is around 130 nm clearly smaller than the

180-200 nm measured in the confocal images. A statistical analysis performed using a t-test further supported this conclusion.

This difference in spatial resolution can also be directly observed in the images of (Supplementary figure 3b); where filaments clearly unresolvable in the confocal image (bottom) can be observed in the decision image (top), and albeit there is some movement between the two images recorded in living cells, it is clear the decision image is sharper than the confocal.

The increase in spatial resolution, compared to the diffraction limited confocal, is caused by the time evolution of the RESOLFT point spread function, which rises from a synergistic action of the ON-switching and read-out illumination. If the two illumination intensities are not close to saturation, then the fluorescence emission is further confined than in conventional microscopy. To further investigate this increase in resolution we decided to do an experiment where we removed the remaining pulsing scheme from the smart RESOLFT, i.e. no donut shaped off-switching and no second Gaussian shaped read-out laser. Furthermore, to ensure every pixel was initiated with the rsFP's in an off state, we added a long Gaussian laser pulse (400μs) at the beginning, followed by a short break to ensure complete separation. The resulting enhanced confocal, and its resulting pulsing scheme is shown in (Supplementary figure 3e) where it can be compared to a conventional confocal (Supplementary figure 3d) recorded sequentially. Both images were recorded with 25 nm pixel size, to ensure similarities with the previous, and followed by smoothing of a Gaussian filter with a radius of 25 nm. Qualitatively it is evident there is a difference in resolution between the two images, confirming the importance of the "two-photon-like" process. The improved spatial resolution of the decision map allows for a more accurate probing since the effective size of the emission spot is even smaller than in conventional confocal microscopy.

Supplementary Note 5

Multi-Image Deconvolution for Smart-RESOLFT

To reconstruct the high-SNR final RESOLFT images we modified a Richardson-Lucy-based multi-image deconvolution algorithm already used in many microscopy techniques, such as, 4Pi[3], stimulated emission depletion (STED)[4] and image scanning (IS) microscopy. Here, we report its basic idea and the main equations to obtain the final algorithm, whose input are the series of $\mathbf{y}_l$ images generated by our time-resolved RESOLFT architecture and the associated point-spread-functions $\mathbf{h}_l$, and the output is an estimation of the protein distribution in the sample.

We assume that for any pixel i=1,...,n of any image $\mathbf{y}_l$ with l=1,...,L, the value $(\mathbf{y}_l)_i$ is the realization of an independent Poisson random variable $Y_{i;l}$ with mean value $(\mathbf{H}_l\mathbf{x})_i$, i.e.,

$$Y_{i;l} \sim \text{Poisson}\{(\mathbf{H}_l\mathbf{x})_i\} \qquad \text{(Eq. 3)}$$

where: (i) $\mathbf{x}$ is a discrete mapping of the protein distribution in the sample (regardless in which state they are, i.e., ON or OFF state); (ii) $\mathbf{H}_l$ is the discrete notation for the convolution operator (linear operator) associated to the l-th PSF $\mathbf{h}_l$. Notably, the different intensities of the images must be encoded in the amplitudes of the PSFs, indeed the total amount of fluorescence photons of each images depends not only on the protein concentration, which is common for all the images, but also on different experimental aspects, such as the pixel dwell-time or the excitation cross-section at the illumination wavelength. Practically, the relation in amplitude between the different PSFs can be obtained comparing the total amount of fluorescent photons collected by the different images (see above).

Thanks to the independence of each random variable $Y_{i;l}$, we can write the probability density distribution as

$$P_Y(\mathbf{y}|\mathbf{x}) = \prod_{l=1}^{L} Y_l = \prod_{l=1}^{L}\prod_{i=1}^{n} Y_{i;l} = \prod_{i,l} \frac{e^{-(\mathbf{H}_l\mathbf{x})_i}((\mathbf{H}_l\mathbf{x})_i)^{(\mathbf{y}_l)_i}}{(\mathbf{y}_l)_i!} \qquad \text{(Eq. 4)}$$

where $\mathbf{y} = \{\mathbf{y}_l\}$.

Since we assume to know the probability density distribution $P_Y(\mathbf{y}|\mathbf{x})$ of the data and the unknown object $\mathbf{x}$ appears as a set of parameters, the problem of restoring $\mathbf{x}$ can be treated as a classical problem of parameters estimation, which is usually solved through the maximum likelihood approach[5].In our case, this approach consists in introducing the likelihood function

$$\mathcal{L}_{\mathbf{y}}^{Y}(\mathbf{x}) = P_Y(\mathbf{y}|\mathbf{x}) \qquad \text{(Eq. 5)}$$

then, the ML-estimate of the unknown object is any object $\mathbf{x}^*$ that maximize the likelihood function with respect to $\mathbf{x}$

$$\mathbf{x}^* = \underset{\mathbf{x}}{\operatorname{argmax}}\, \mathcal{L}_{\mathbf{y}}^{Y}(\mathbf{x}) \qquad \text{(Eq. 6)}$$

This maximization problem can be transformed into a minimization problem by considering the negative logarithm of the likelihood function[5], which leads to the minimization of the functional

$$J(\mathbf{y};\mathbf{x}) = \sum_{i,l}\left((\mathbf{y}_l)_i \ln\frac{(\mathbf{y}_l)_i}{(\phi_l\mathbf{H}_l\mathbf{x})_i} + (\phi_l\mathbf{H}_l\mathbf{x})_i - (\mathbf{y}_l)_i\right) \qquad \text{(Eq. 7)}$$

which is the well-known Kullback-Leibler (KL) divergence of the vector $\mathbf{H}_l\mathbf{x}$ from the vector $\mathbf{y}$. To minimize the KL divergence under the non-negativity constraint $\mathbf{x}>0$ we used the slit-gradient-method (SGM)[6], which leads to the following iterative algorithm

$$\mathbf{x}^{k+1} = \mathbf{x}^k \left(\sum_{l=1}^{L} w_l \mathbf{H}_l^T \frac{\mathbf{y}_l}{\mathbf{H}_l \mathbf{x}} \right) \quad \text{(Eq. 8)}$$

where $\mathbf{H}^\mathrm{T}$ is the transpose of the convolution operator $\mathbf{H}$, and w_l are factors which encode the different intensities of the images and thus the amplitudes of the PSFs

$$w_l = \sum_n \mathbf{h}_l(n). \quad \text{(Eq. 9)}$$

For the sake of simplicity, we move to a vector notation

$$\mathbf{x}^{k+1} = \mathbf{x}^k \left(\sum_{l=1}^{L} w_l \mathbf{h}_l \star \frac{\mathbf{y}_l}{\mathbf{h}_l * \mathbf{x}} \right), \quad \text{(Eq. 10)}$$

where * denotes the convolution operation, implemented through fast Fourier transform. In this work the algorithm is initialized with a uniform image, i.e., $\mathbf{x}^0 = \mathbf{1}$, and only a few number of iterations are performed.

Supplementary Note 6

Point-Spread-Function Modeling

The PSFs for the deconvolution algorithm are obtained from the model described above. In particular, we used the spatial distribution of fluorescence collected at the end of each steps. The calculation needs as input the optical parameters and spectroscopy parameters. The illumination and emission wavelengths, the objective lens numerical aperture, and the pixel-size allow us to calculate the different focal intensity distributions[7]. The population of the ON state are calculated for a given $I_{\text{sat\_ON}}$, I_{satRead1}, and $I_{\text{sat\_OFF}}$. In particular, we used low $I_{\text{sat\_ON}}$ and I_{satRead1} values to maintain a linear relation between intensity and activated/deactivated population. On the other side, $I_{\text{sat\_OFF}}$ is based on the dataset. To estimate the value of $I_{\text{sat\_OFF}}$ we choose a value able to produce an effective PSF of the RESOLFT image in agreement with the FWHM of the intensity profiles of sub-diffraction sized structures in the image. We finally assume linearity between the emitted fluorescence and the population of the ON state. Given the PSFs for the different images, we normalize their integral to 1 and we multiply each PSF $\mathbf{h}_l$ for a weigh-factor wf_l proportional to the total intensity of the relative real image $\mathbf{y}_l$

$$wf_l = \frac{\sum_n \mathbf{y}_l}{max(\sum_n \mathbf{y}_i | i = 1, \dots, L)} \,. \qquad \text{(Eq. 11)}$$

Supplementary Note 7

Dynamical investigation of peroxisomes with smart RESOLFT

A detailed comparison between confocal, smart RESOLFT, and regular RESOLFT for the dynamical measurement of the peroxisomes is presented in (Supplementary figure 5a-c). The frame time for these are 1,5s, 1,8s and 5,3s, note the frame time is slower compared to (Figure 5a-b) due to larger field of views. When imaging moving objects, such as the peroxisomes, with a point scanning system, a particular attention has to be paid to avoid scanning artifacts. These can occur if the object, or in this case the peroxisome, moving during the time it takes to scan a single line, and as such is highly dependent on the frame time. With the smart RESOLFT, we approached the speed of the confocal (Supplementary figure 5a'-c'), and it is clearly evident that the smart RESOLFT (and the confocal) shows fewer scanning artifacts compared to the conventional RESOLFT. However, when measuring lines profiles across the peroxisomes (Supplementary figure5d-g) it is clear that we still maintain the resolution and as such we gain the resolution of RESOLFT at the speed of a confocal. It is worth remembering that we aimed at a resolution of about 100 nm by decreasing the off time to 250 µs (Supplementary figure 1b), since it was deemed sufficient for this study, and meant we could increase the speed even further. Had we instead used smart RESOLFT with the full 500 µs off the frame time would have been 2,8s for the smart RESOLFT instead of the 1.8s.

During the tracking presented in the main article (Figure 5a-b) we observed several events of fusion and fission, a few examples of this is shown in (Supplementary figure 5h-i), and indeed in some of the frames we still observe scanning artifacts, however they are never severe enough to actually prohibit the detection of the position of the peroxisomes or determine their interactions. One of the strengths of super resolution, if not the definition, is the ability to

separate objects close to each other, in this study it allowed us to clearly separate when two peroxisomes where interacting or simply getting in proximity, an example of the latter is shown (Supplementary figure 5j) with a comparison with simulated confocal data, generated by a Gaussian filter. It is clear that the confocal data would lead to the wrong conclusion of a fusion event, whereas the smart RESOLFT clearly shows the separation of the peroxisomes through all three frames.

In the data presented in the main paper we show that we can perform particle tracking on the individual peroxisomes at the time series shown in (Figure 5a-b), as mentioned previously the frame time is highly dependent on the structures in the frame and varies during the acquisition of the time series. The instantaneous frame rate during the 500 frames is shown in (Supplementary figure 5k), and ranges from 2-4,5 Hz. The particle tracking was carried out on the first 100 frames, and measured velocity is plotted (Supplementary figure 5l) together with a half normal fit, with the 435 steps we measure a velocity of 0,44±0,33 µm/s (The expectation value ± the square root of the variance).

Supplementary Note 8: Dynamical measurement of mitochondrial outer membrane

In the main paper (Figure 5e-f) we followed the dynamical movement of the mitochondria outer membrane by performing an xt-scan across a single mitochondrion. Overall we recorded 9600 lines at an average rate of 31 Hz, the start and the end of the series can be seen in (Supplementary figure 6a,d) with a line profile at both location (Supplementary figure 6c) showing we still have approximately half the signal left after the 9600 measurements. During the measurement, we observed the spontaneous fission and fusion of the mitochondria, and one example of such event is shown in (Supplementary figure 6b)

Drawing line profiles across this event (Supplementary figure 6c) allows us to follow the outer membrane of the mitochondria and also observe the appearance of the extra membrane formed during the fission (Supplementary figure 6c,III).

Supplementary Note 9

Smart RESOLFT set-up

Smart RESOLFT microscopy was carried out using a custom-built setup as illustrated in the (Supplementary figure 7). Three lasers from Cobolt AB one at 405 nm and two at 488 nm (Cobolt AB 06-MLD, Solna, Sweden) were chosen for modulable in time with microsecond accuracy. The OFF-switching laser passed through a Glen-Thompson prism to ensure linear polarized light, and a phase plate (VPP-1, RPC Photonics, NY, USA) to ensure the 0-2pi phase shift required for the OFF-switching doughnut shaped light pattern. The OFF-switching beam was then combined with the 488 nm read-out laser by a polarizing beam splitter. The ON-switching laser (405 nm) was combined with the two other lasers using a 556 SP (FF556-Sdi01, Semrock, NY, USA), which also reflected the 405 nm. The combined laser beams where passed a set of galvo mirror scanners for x and y (Cambridge technology, MA, USA) and passed through a scan lens and a tube lens, before arriving at the objective lens, a 100x oil STED white, NA 1.4 (Leica, Wetzlar, Germany), which was mounted on a Leica microscope stand. The stage was custom-designed and mounted directly onto the objective lens to minimize any drift or vibration, with a built-in piezo for z-positioning (PI, Karlsruhe, Germany). The emission light was de-scanned and decoupled using a 488 LP (ZT488rdc, Chroma, VT, USA), and a 535 BP filter (E535/70M, Chroma, VT, USA). The fluorescence was then fiber coupled (105 μm), the fiber core act as a pinhole. The emission light was recorded using an SPAD (MPD, Bolzano,

Italy). The xy-galvo's, the z-piezo and the pulsing scheme of the lasers was controlled using carma, a custom electronic control system for scanning microscopy, based on a FPGA PCIe-7852R card (National Instruments).

Supplementary Note 10

Photo-toxicity in RESOLFT and STED

In general, phototoxicity is a complex manner, which can vary depending on peak intensity, overall light doses as well as wavelength, cell types and even specific structures inside the same cell. On this line we provide below values of these imaging parameters used today in RESOLFT and STED live cell imaging.

Both STED and RESOLFT microscopy can be implemented with a doughnut shape OFF pattern, which features an average power measured at the pupil of about 50-190 mW (STED) or 1-10 uW (RESOLFT) respectively. In both techniques the pixel dwell time depend very much on the number and brightness of the fluorescent molecules tagging the structure of interest as in confocal or two-photon microscopy.

Example of published live cell imaging studies using STED microscopy reported:

- Mash et al.[8] used pulse STED at 775 nm, 53-66 mW of average STED power, 30 us dwell time. Imaging of bright post synaptic densities in neurons with stated spatial resolution of about 80-95 nm. The authors specified that to obtain spatial resolution of about 53 nm nm they needed to increase the STED power to 169 mW. Peak power is about 20 times higher considering the STED pulse width of 600 ps and 80MHz repetition rate. The total light dose is 3810-12900 J cm^{-2}.

- Bottanelli et al.[9] used pulse STED at 775 nm, 140 mW of average STED power, peak power is about 20 times higher (600 ps pulse duration for 80 MHz) applied for 1.8 us as total pixel dwell time. Imaging of organelles in cell culture. The light dose results of 642 J cm^{-2}.

- Hein et al.[10] used CW STED at 594 nm, 112 mW of averaged STED power, which resulted in 62 MW/cm2 applied for a total pixel time of 0.05 ms. Imaging of the ER labeled with Citrine as bright YFP variant. The total light dose is 3100 J cm^{-2}.

Peak intensity. Considering the above-mentioned values for STED we find that the RESOLFT peak intensity (14-19 kW cm^{-2}) is orders of magnitude lower than STED (89-350 MW cm^{-2}). Therefore, RESOLFT has the potential to reduce photo-bleaching by high order photon interaction, which is known to trigger ROS formation, DNA damage and plasma formation.

RESOLFT imaging applied to green emitting rsFPs such as rsEGFP2 and DronpaM159T, as in this work, requires an average power of 1-10 uW applied for 0.3-0.5 ms. The intensities are 14-19 kW cm^{-2}. Total light dose of 7-9.5 J cm^{-2} for OFF switching at 488 nm.

Wavelength. STED as well as RESOLFT can be applied at different wavelength depending on the choice of the fluorescent molecule. In this work smart RESOLFT was applied to rsEGFP2 and other green-emitting negative switchers, which need 405 nm and 488 nm for switching ON and OFF and excitation. The live cell compatibility of rsEGFP2 imaging intensities has been previously demonstrated (Wäldchen et al.[11]; Masullo et al.[12], Supplementary Information, section 7).

Wäldchen et al.[11] did a very detailed study on 405 nm irradiation and the impact on cell viability. They concluded that the damage threshold for the light dose at 405 nm was ~50 J cm−2 in a wide-field homogenous illumination. To be noticed that this light dose is much higher than the one required in RESOLFT imaging. As shown in table 1 in the Supplementary information,

intensities at 405 nm ranging from 2-3 kW/cm^2 with a pulse ranging from 30 to 70 us are used for RESOLFT imaging, which lead to 0.09-0.21 J cm^{-2}.

Considering the reported intensities values the sample could handle around 200 pulses of the maximum dose before being at risk of damage. In a similar way we can estimate that the light dose at 488 nm delivered during RESOLFT imaging for both the OFF-switching and read-out light. They are well below the ~48 kJ cm^{-2} also reported in Wäldchen et al work.

Also, the calculations made are in well agreement with the cell viability experiments performed on negative switcher[12].

Finally, it is important to consider that the concept of smart RESOLFT can also be applied to red reversible switchable proteins such as the new rsFusionRed requiring only 510 and 590 nm and not UV–Vis light. Thus, further minimizing UV-Vis induced photo-toxicity and DNA damage as demonstrated by the experiment illustrated in the supplementary material and Supplementary figure 12 of Pennacchietti et al. [13].

Image	Laser intensity (kW/cm^2)			Laser timings and pulsing scheme					
	405 nm activation	488 nm off switching	488 nm Read-out	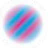	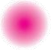	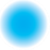	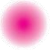	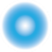	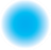
Fig 2a	3,1	13,8	12,2	40			50	500	50
Fig 2b	3,1	13,8	12,2		20	50	50	500	50
Fig 2c	3,1	13,8	12,2		50	40		500	50
Fig 2d	3,1	18,5	16,1				50	500	50
Fig 3	3,1	18,5	16,1		20	50	50	500	50
Fig 4a	3,1	18,5	16,1		20	50	50	500	50
Fig 4b	3,1	18,5	16,1		20	50	50	500	50
Fig 5a+b (500 frames/peroxizomes)	3,1	18,5	16,1	40			40	250	40
Fig5c-d (5 frames)	2,4	13,8	15,7	40			50	500	50
Fig5e (9600 line scans)	2,4	13,8	15,7	40			50	500	50
Fig 6 (worm) (40 stacks of 10 slice)	3,1	8,84	12,2		20	50	50	500	50

Supplementary Table 1 Table of all the laser powers and pulsing schemes and timing used in the different figures.

Image	Skipped pixels (%)	Dosage		
		Regular RESOLFT (mJ)	Fast RESOFT (mJ)	Saved(%)
Fig 2a	64	0,269	0,108	60
Fig 2b	70	0,269	0,0933	65
Fig 2c	51	0,269	0,145	46
Fig 2d	28	0,361	0,276	22
Fig 3	26	0,445	0,349	22
Fig 4a	94	12,6	1,33	90
Fig 4b	87	3,56	0,622	83
Fig 5a+b (500frames/peroxizomes)	88	4,49	0,892	80
Fig 5c+d (6 frames)	62	2,798	1,20	57
Fig 5e (9600 line scans)	42	2,865	1,803	37
Fig 6 (worm) (40 stacks of 10 slice)	79	31,3	8,76	72

Supplementary Table 2 Table of the skipped pixel and resulting lower photo dosage for the different figures.

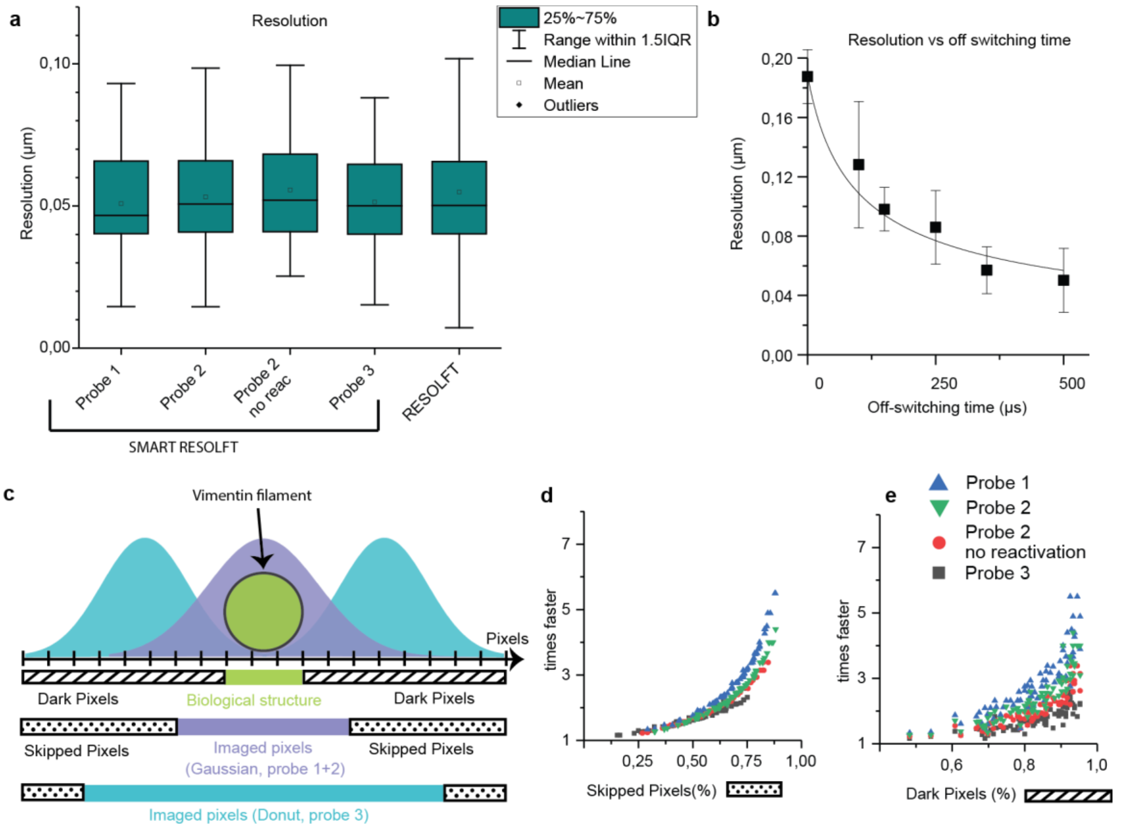

Supplementary Figure 1 Measurements of the spatial resolution of regular and smart RESOLFT microscopy performed with four different probe types. (**a**) The graph shows the distribution of measured FWHM of single vimentin filament widths (Lorentzian fits to line profile averaged over 3 pixels). The median values are 46 nm, 51 nm, 52 nm, 50 nm and 50 nm for the probe 1, 2, 2 no reactivation, 3 and regular RESOLFT respectively. Each distribution is made of at least 60 different measured line profiles. (**b**) The graph shows the resolution as a function of the off time, the resolution increases with increasing OFF-switching duration. The light intensities used for these measurements were: 3.1 , 18.5 , 12.2 kW/cm^2 for ON-switching, OFF-switching and read-out respectively. Both the ON-switching and the read-out were kept the same at 50µs for all the experiments. **(c)** The scheme represents a cross section of a vimentin filament in green illuminated with focused light of different shape. In light-blue and violet colors are represented the Gaussian-shaped and a doughnut-shaped probe illumination. Dark

pixels represent region in the sample that contain no structures while skipped pixel are the one that smart RESOLFT identifies as containing no structures and therefore where no RESOLFT imaging scheme is applied. The doughnut shaped illumination leads to the acquisition of more "dark" pixels compared to the Gaussian-shaped beam due to the slightly larger size as quantified in graphs (**d-e**). In graph (**d**) the increase in recording speed is compared to the percentage of skipped pixel in the measured images. A clear ranking of the 4 different probe types emerges with the fastest being probe type 1 > probe type 2 > probe type 2 – no additional ON-switching > probe type 3. This ranking is consistent with the decision time for the different probes (40µs, 70 µs, 90 µs, 150 µs). In graph (**e**) the increase in recording speed is measured according to the actual biological structure, which is identified in regular RESOLFT images by applying a threshold mask to separate the structure form the background.

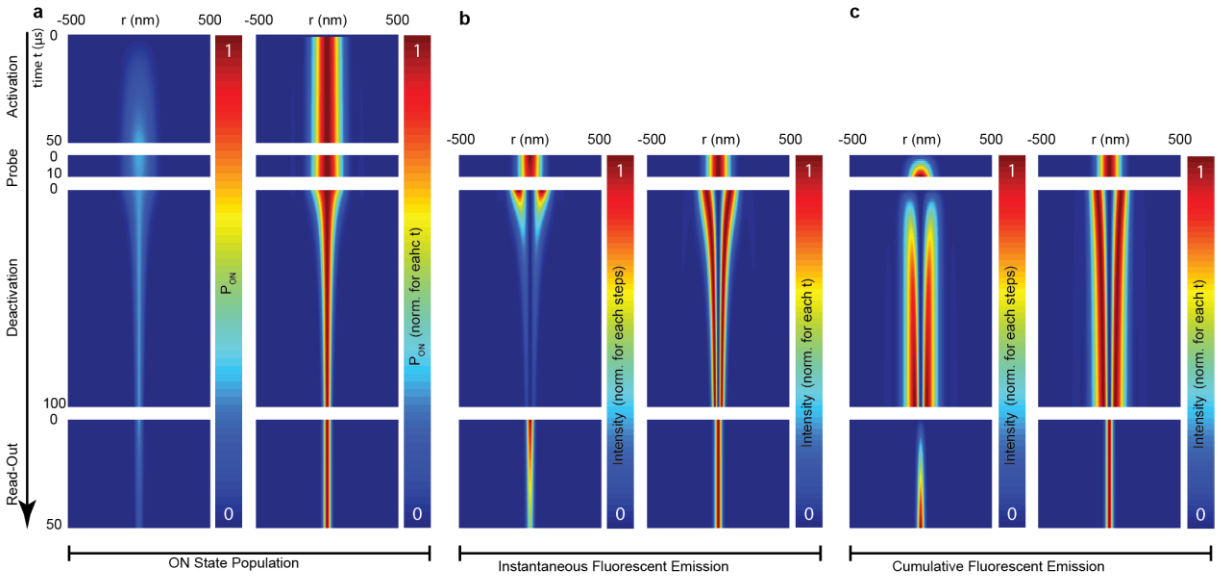

Supplementary Figure 2 Temporal and spatial evolution of the distribution of the ON state population and of the PSF across the whole pixel RESOLFT cycle. (a) Temporal and spatial evolution of the population of the ON state during the four different steps (ON-switching, Probe, OFF-switching, and Read-Out). The right column depicts the population normalized at each time-position. (b) Spatial and temporal distribution of the fluorescence flux, i.e., instant PSFs, for the four different steps. The instantaneous PSFs are normalized with respect to the maximum value achieved during the respective step. The right column depicts the flux normalized at each time position. (c) Cumulative PSF, i.e., the signal is integrated during the time-course of the pixel. The right column depicts the cumulative PSF normalized at each time-position.

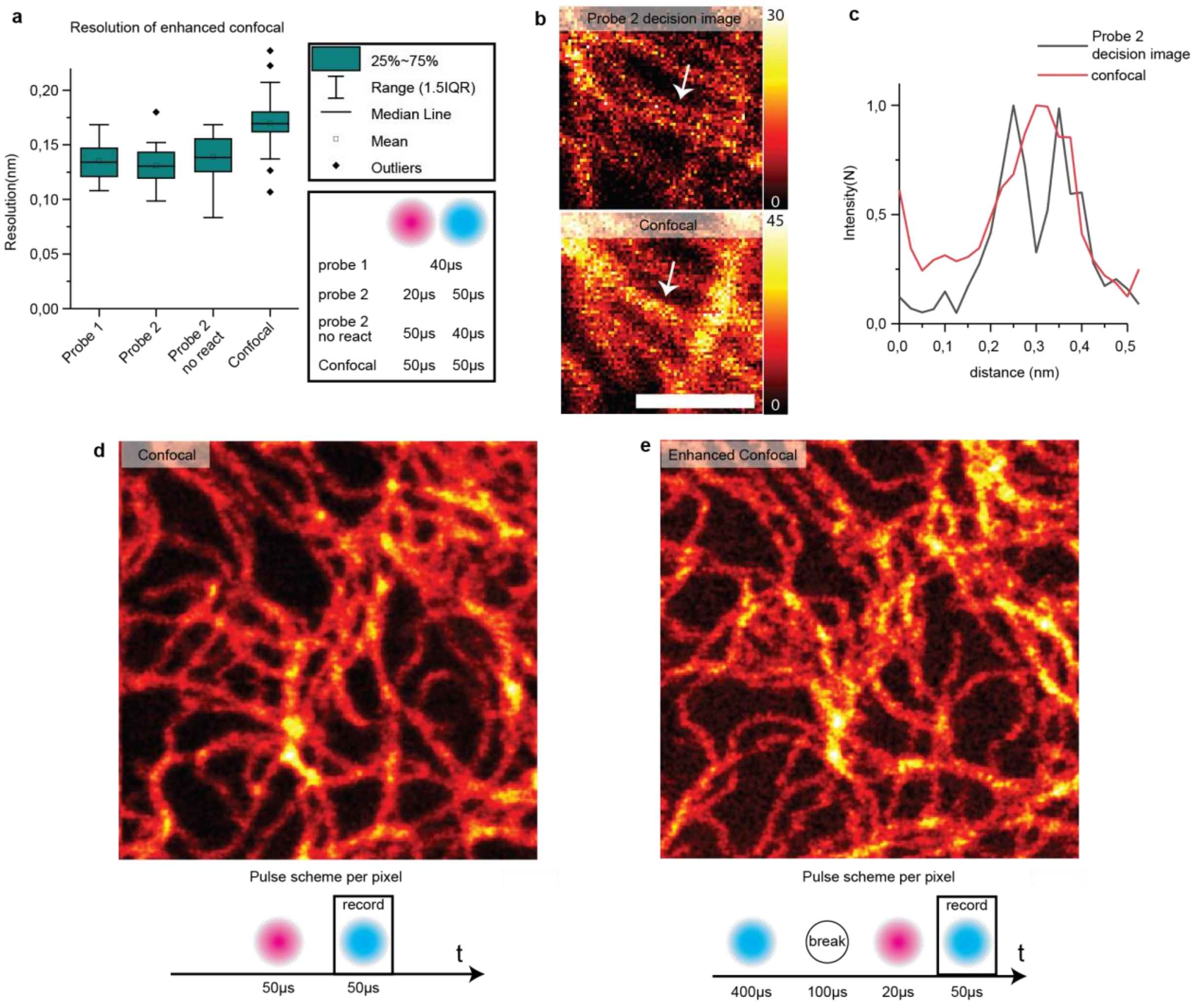

Supplementary Figure 3 The fluorescence photons generated during the probe illumination by the rsFPs form an image coined enhanced confocal. The spatial resolution of the enhanced confocal images is shown in the graph (**a**) for the different probe types and as a comparison a conventional confocal. Vimentin filament widths were measured (line profile averaged over 3 pixels) and fitted (Lorentzian fits). The FWHM of the fit is plotted in the graph and gives rise to distribution with mean values at 139 nm, 140 nm, 130 nm and 170 nm respectively for the probe 1, 2, 2 with no additional ON-switching and confocal respectively. A t-test shows a significant difference between the resolution in the confocal and the other. However, no significant difference is found for the different probing schemes. (**b**) A

representative enhanced confocal image, acquired with probe type 2 mode, is compared to a conventional confocal recording of the same region of the cell. The difference is highlighted in the graph (**c**) plotting the intensities of the line profile measured across two vimentin filaments, which are indistinguishable in the confocal recording. (**d-e**) To further investigate whether this increase in resolution was caused by "two-photon-like" process we recorded an enhanced confocal (**e**) which lacked the remaining pulses (i.e. the donut shaped and conventional read-out), to further ensure we started every pixel from a complete off-state we also included a long off-switching (400µs) at the start of the pixel. This can qualitatively be compared to a conventional confocal (**d**). The images were recorded with 25 nm pixel and smoothed with a Gaussian filter with a radius of 25 nm. Scale bars 1 µm.

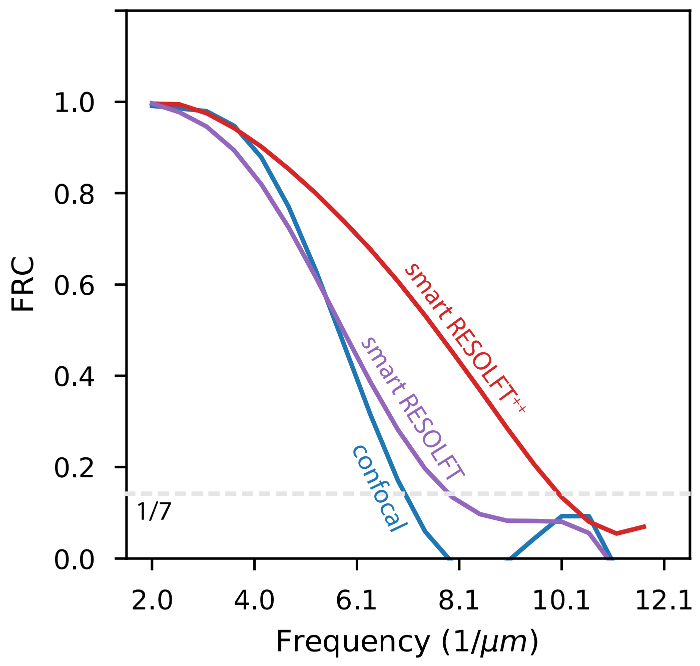

Supplementary Figure 4 Fourier ring correlation (FRC) analysis of smart RESOLFT. The FRC analysis shows the enhancement in resolution of smart RESOLFT microscopy compare to confocal microscopy. However, such enhancement in resolution (123 nm vs 144 nm) is weaker than the enhancement showed by measuring the resolution using the more conventional line intensity profile criteria. This is fully expected, since it is well known that the FRC-based resolution metric is very sensitive to SNR. Tortarolo et al.[14] shows that the differences in estimating the resolution with the FRC metric or with the more conventional intensity profile metric can be substantial (200 nm instead of 100 nm) for low SNR imaging. Very important, the FRC analysis on the reconstructed smart RESOLFT++ image show a clear resolution improvement, since the SNR improves. In conclusion the FRC analysis fully confirm the sub-100 nm resolution provided by our proposed approach.

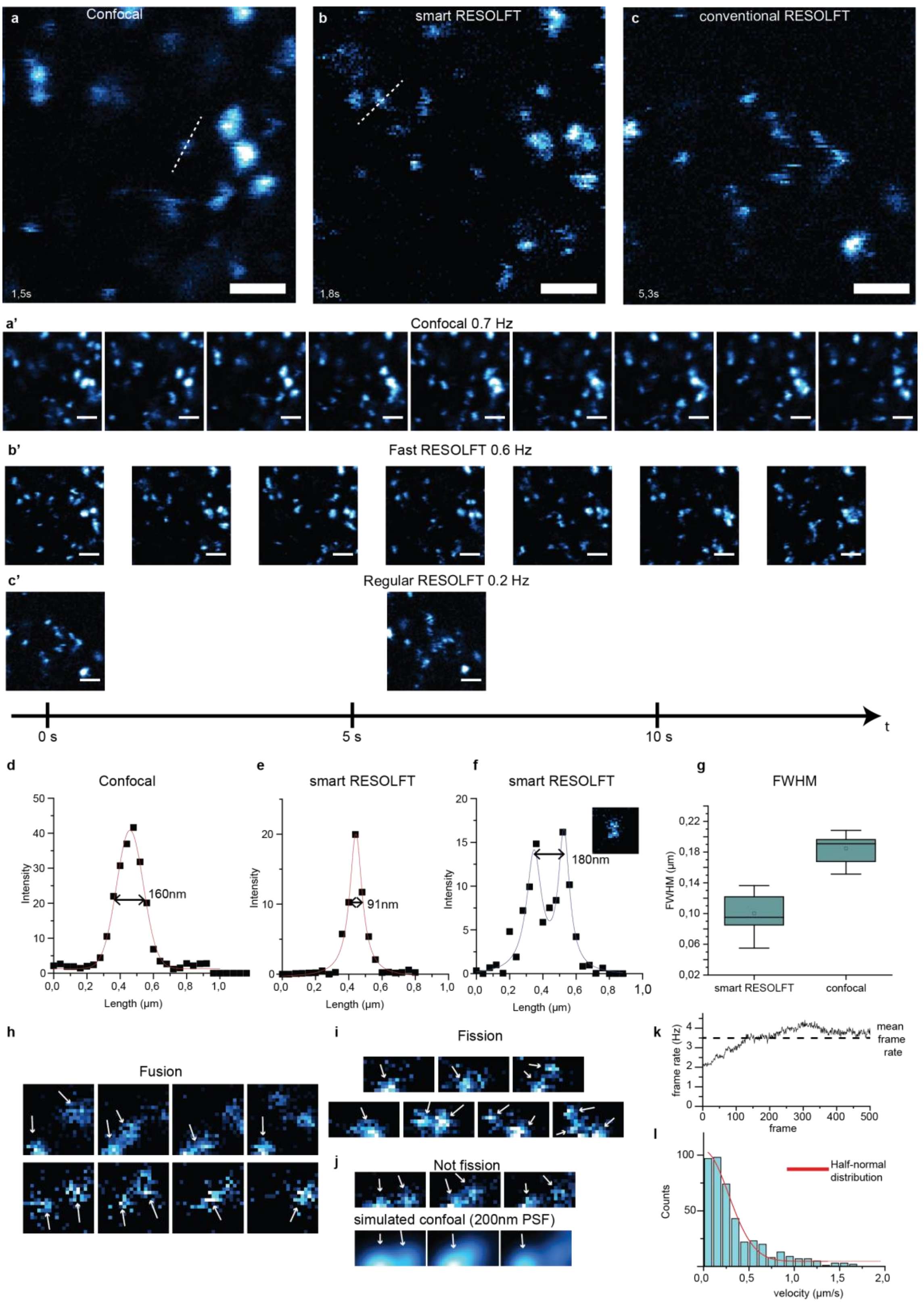

Supplementary Figure 5 Peroxisomes dynamics recorded in the confocal, conventional RESOLFT and smart RESOLFT modes. (**a-c**) examples of images recorded with a confocal, a smart RESOLFT and a conventional RESOLFT microscope. The size of the frame is larger

than the one recorded in **Figure 5a-b**, which explains the lower frame rate of 0.7 Hz, 0.6 Hz and 0.2 Hz. The two RESOLFT techniques show higher spatial information. However, the conventional RESOLFT suffers from scanning artifacts, which decreased in the confocal and the smart RESOLFT the scanning artifacts. It is worth noting that the scan speed of the smart RESOLFT approaches the scan speed of the confocal, as is illustrated in (**a'-c'**). Scale bars are 1 µm. In (**d-f**) are shown selected line profiles from the confocal (**d**) and the smart RESOLFT (**e,f**) highlighting the higher spatial resolution in the RESOLFT image. A statistic of several (N=15) line profiles is shown (**g**) providing 100 nm of FWHM across the peroxisomes. Care was taken when measuring the FWHM to not measure peroxisomes where scanning artifacts were present. All line profiles were an average of 3 lines. The OFF switching time was shortened at 250 us since no smaller structures need to be rsolved. (**h,i**) Shows examples of fusion and fission processes observed in the recorded time series of **Figure 5a-b**, highlighting the RESOLFT imaging capability to distinguish when fusion/fission processes occur and when two peroxisomes are merely in proximity, which would have been missed in (**j**) confocal representation obtained by smoothing the smart RESOLFT data with a 200 nm diameter Gaussian filter. The width of the images in (**h-j**) are 800 nm. (**k**) graph of the frame rate for each individual frame, the mean value for all 500 frames is 3.5 Hz. (**l**) distribution of measured velocities in the peroxisome tracking in **Figure 5**, and a half-normal fit to the data.

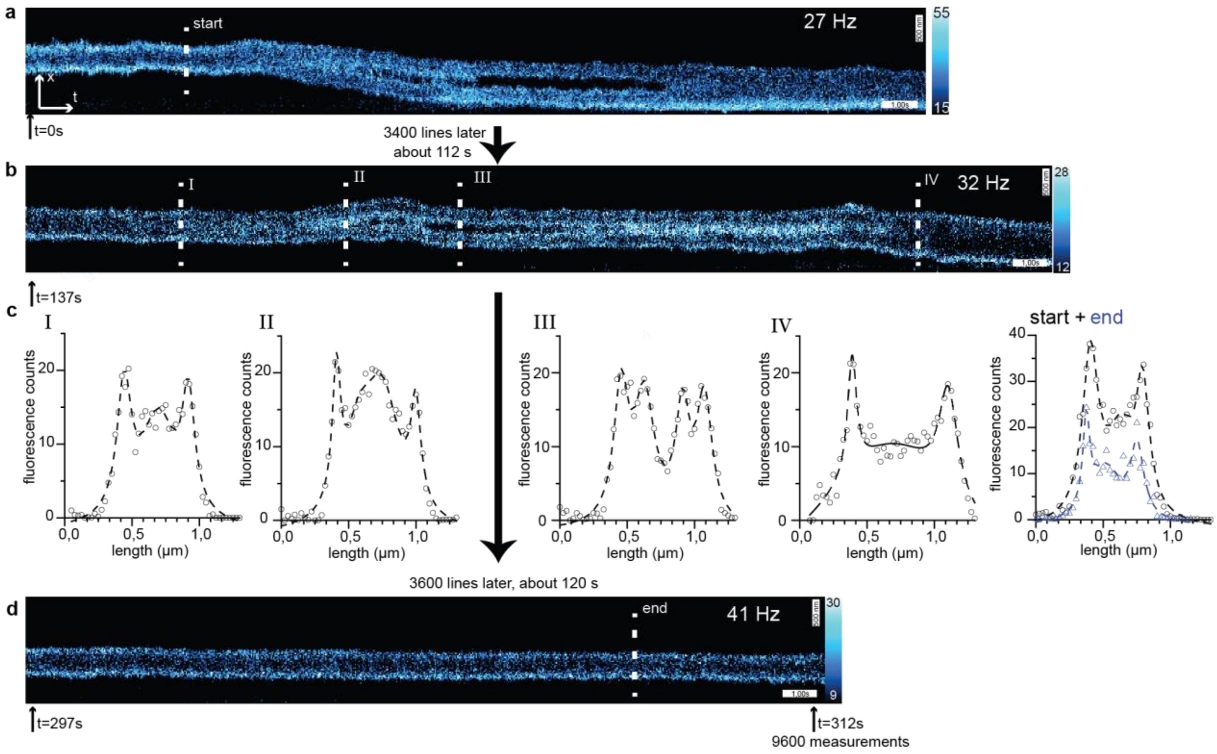

Supplementary Figure 6 Mitochondrial outer membrane dynamics. Line scan of a mitochondrion cross section has been acquired continuously at 27-40 Hz since the smart RESOLFT adapts the recording speed according to the structure (**a, b, d**). We acquired 9600 lines in 312 s resulting in an average speed of 31 Hz. Over time we could detect splitting and fusion events. (**c**) The line profiles before (I), during (II-III) and after (IV) splitting are shown. Overall 4 events were observed during the recording. The line profiles are measured in the marked and labeled lines shown in the images a and b and are an average of 3 lines. The data shown in a, b and d are smart RESOLFT raw data. Less than 50% of the initial fluorescent signal was lost after thousands of time points of observation, as shown in the graph representing the start (black) and end (blue) line profile, highlighting the rsFPs minimal switching fatigue. Scale bars in the vertical (x) direction are 500 nm, in the horizontal (t) they are 1 s.

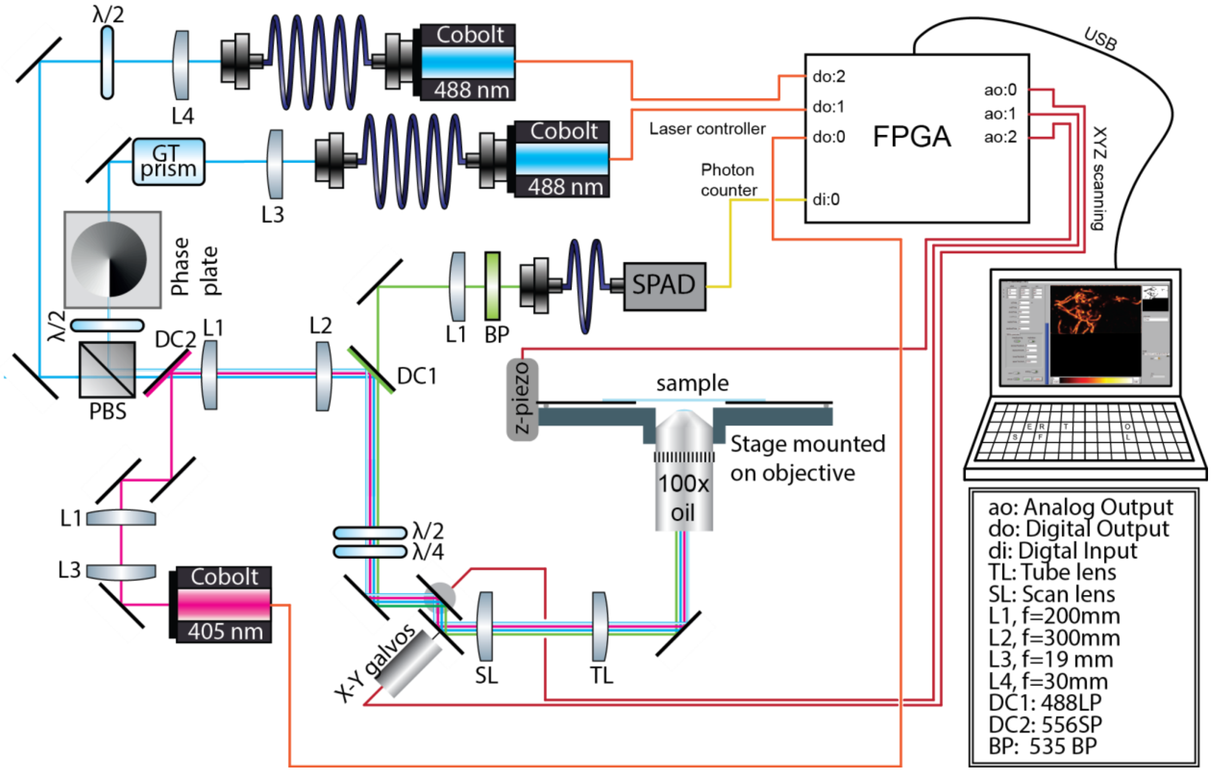

Supplementary Figure 7 Smart RESOLFT imaging platform. The optical and electric components of the setup are represented and described in the figure. L= lenses. GT= Glan Thompson. BP= band pass filter. PBS= polarized beam splitter. λ/2 and λ/4 retarder waveplates. DC= dichroic filter.

Supplementary References

1. Galiani S, *et al.* Super-resolution Microscopy Reveals Compartmentalization of Peroxisomal Membrane Proteins. *The Journal of biological chemistry* **291**, 16948-16962 (2016).

2. Leutenegger M, Gosch M, Perentes A, Hoffmann P, Martin OJF, Lasser T. Confining the sampling volume for Fluorescence Correlation Spectroscopy using a sub-wavelength sized aperture. *Opt Express* **14**, 956-969 (2006).

3. Vicidomini G, Schmidt R, Egner A, Hell SW, Schonle A. Automatic deconvolution in 4Pi-microscopy with variable phase. *Opt Express* **18**, 10154-10167 (2010).

4. Castello M, Diaspro A, Vicidomini G. Multi-images deconvolution improves signal-to-noise ratio on gated stimulated emission depletion microscopy. *Appl Phys Lett* **105**, (2014).

5. Zanella R, Boccacci P, Zanni L, Bertero M. Efficient gradient projection methods for edge-preserving removal of Poisson noise. *Inverse Probl* **25**, (2009).

6. Vicidomini G, Boccacci P, Diaspro A, Bertero M. Application of the split-gradient method to 3D image deconvolution in fluorescence microscopy. *J Microsc-Oxford* **234**, 47-61 (2009).

7. Leutenegger M, Gosch M, Perentes A, Hoffmann P, Martin OJ, Lasser T. Confining the sampling volume for Fluorescence Correlation Spectroscopy using a sub-wavelength sized aperture. *Optics express* **14**, 956-969 (2006).

8. Masch JM, *et al.* Robust nanoscopy of a synaptic protein in living mice by organic-fluorophore labeling. *Proceedings of the National Academy of Sciences of the United States of America* **115**, E8047-E8056 (2018).

9. Bottanelli F, *et al.* Two-colour live-cell nanoscale imaging of intracellular targets. *Nature communications* **7**, (2016).

10. Hein B, Willig KI, Hell SW. Stimulated emission depletion (STED) nanoscopy of a fluorescent protein-labeled organelle inside a living cell. *Proceedings of the National Academy of Sciences of the United States of America* **105**, 14271-14276 (2008).

11. Waldchen S, Lehmann J, Klein T, van de Linde S, Sauer M. Light-induced cell damage in live-cell super-resolution microscopy. *Scientific reports* **5**, 15348 (2015).

12. Masullo LA, Boden A, Pennacchietti F, Coceano G, Ratz M, Testa I. Enhanced photon collection enables four dimensional fluorescence nanoscopy of living systems. *Nature communications* **9**, (2018).

13. Pennacchietti F, *et al.* Fast reversibly photoswitching red fluorescent proteins for live-cell RESOLFT nanoscopy. *Nature methods* **15**, 601-604 (2018).

14. Tortarolo G, Castello M, Diaspro A, Koho S, Vicidomini G. Evaluating image resolution in stimulated emission depletion microscopy. *Optica* **5**, 32-35 (2018).
